# Rheological Behavior, Filament Stability, and Microstructure of an Extrusion-Processable Kefiran–PG Formulation

**DOI:** 10.3390/polym18060732

**Published:** 2026-03-17

**Authors:** Elisa Capuana, Emmanuel Fortunato Gulino, Roberto Scaffaro, Valerio Brucato, Vincenzo La Carrubba

**Affiliations:** Department of Engineering, University of Palermo, Viale Delle Scienze, Ed. 8, 90128 Palermo, Italyroberto.scaffaro@unipa.it (R.S.); vincenzo.lacarrubba@unipa.it (V.L.C.)

**Keywords:** kefiran, extrusion-based additive manufacturing, time-dependent viscoelasticity, physical network formation, porous polymer microstructure

## Abstract

Microbial polysaccharides are attracting increasing interest as water-processable polymers for extrusion-based additive manufacturing due to their ability to form physically stabilized networks without covalent cross-linking. In this study, a kefiran–propylene glycol (PG) formulation was developed to investigate whether time-dependent supramolecular reorganization can be exploited to control print fidelity. Extrusion performance was assessed through quantitative filament collapse analysis, while rheological behavior was characterized by oscillatory strain, frequency, and time sweep measurements. Filaments printed 5 min after PG addition showed pronounced sagging (*δ*/(*L*/2) ≈ 0.35 at the largest spans), whereas after 15 min the normalized deflection decreased below 0.03, indicating a marked improvement in self-supporting capability. Time sweep experiments revealed a continuous increase in storage modulus from ~100 to ~1200 Pa over 1800 s, consistent with progressive viscoelastic stiffening. Freeze-dried constructs exhibited an interconnected porous architecture with a predominant pore population between 6 and 20 µm and an apparent porosity of 60.9 ± 1.2%. Upon rehydration at 37 °C, samples swelled to ~350% within 5 h and showed gradual mass loss over 56 days while remaining intact. ATR–FTIR confirmed the preservation of the polysaccharide backbone without evidence of new covalent functionalities. Extrusion fidelity is therefore governed by progressive supramolecular consolidation within a physically assembled network, rather than by any form of chemical cross-linking.

## 1. Introduction

Polymeric materials derived from renewable biomass are increasingly investigated as alternatives to conventional petrochemical systems, particularly in the context of water-based processing and physically structured polymer networks [1,2,3]. In recent years, bio-based polymers have been adopted in applications ranging from packaging [4] and biomedical devices [5] to agriculture and consumer products [6]. Synthetic aliphatic polyesters such as polycaprolactone (PCL) and polylactic acid (PLA) [7], as well as microbially produced polyesters including polyhydroxyalkanoates, are widely studied due to their defined chemistry and established processing routes [8]. However, many of these systems require elevated temperatures, organic solvents, or reactive modifications to achieve adequate processability and structural stabilization [9,10,11].

For applications involving extrusion-based shaping and additive manufacturing, polymer systems that can be processed in aqueous media and that stabilize through reversible, non-covalent interactions are of particular interest. In this context, natural polysaccharides represent an attractive class of materials due to their structural diversity and intrinsic ability to form physically associated networks [12]. Among them, marine and microbial polysaccharides have received attention as scalable and reproducible polymer sources [13,14]. Nevertheless, only a limited subset has been systematically evaluated in terms of extrusion-specific rheology and filament-level behavior.

Alginate is one of the most extensively studied polysaccharides for extrusion-based processing, typically relying on ionic cross-linking to achieve post-deposition stabilization [15,16]. Various strategies, including controlled ion release, internal gelation, and dual cross-linking approaches, have been developed to tailor its processing window [17,18]. These approaches demonstrate the feasibility of polysaccharide-based extrusion; however, they also highlight the importance of balancing flow behavior during deposition with controlled structural consolidation afterward. In this respect, polymer systems capable of forming stable networks through composition- and time-dependent physical interactions, without external gelling steps, are of considerable interest for extrusion-based shaping.

Microbial polysaccharides constitute a particularly relevant subgroup due to their production through controlled fermentation processes, which enables reproducible material properties and scalable manufacturing [19,20]. Despite these advantages, detailed investigations of their extrusion behavior (especially at the filament scale) remain limited. In many cases, studies focus on bulk or cast hydrogels, providing only indirect insight into flow during extrusion, filament formation, gravitational sagging, and time-dependent structural evolution after deposition [21,22].

Kefiran is an extracellular polysaccharide produced during kefir grain fermentation by a microbial consortium dominated by *Lentilactobacillus kefiranofaciens* [23]. In aqueous environments, kefiran behaves as a flexible, water-soluble polymer capable of forming three-dimensional physical networks driven by hydrogen bonding and weak intermolecular interactions [24,25]. Its structure is known to depend on concentration and resting time, proceeding through progressive chain association rather than ion-mediated cross-linking [26,27]. These characteristics suggest potential suitability for extrusion-based processing, where both flow under shear and gradual structural consolidation after deposition must be controlled to preserve filament geometry [28,29].

Despite reports on kefiran hydrogels and bulk gelation behavior, a systematic evaluation of kefiran-based formulations in the context of extrusion processing is lacking. In particular, quantitative analyses of filament stability, time-dependent viscoelastic evolution following formulation preparation, and the relationship between rheological consolidation and extrusion performance have not been comprehensively addressed [25,30]. Moreover, the microstructural features that develop after extrusion and drying, as well as swelling and mass variation under aqueous conditions, remain insufficiently characterized for extrusion-processed kefiran systems.

In this work, we develop and characterize a kefiran-based formulation tailored for extrusion-based additive manufacturing. Propylene glycol (PG) is introduced as a low-molecular-weight processing aid to modulate intermolecular interactions while maintaining fully aqueous processing and avoiding chemical cross-linking reactions. At the molecular level, PG is anticipated to compete with water molecules for hydrogen-bonding sites along the kefiran backbone [31,32], thereby disrupting excessive chain–chain association that would otherwise impede flow during extrusion. This plasticizing effect [33] allows for controlled modulation of the hydrogen-bonding equilibria: PG can transiently screen direct kefiran–kefiran interactions during shear flow while permitting progressive network consolidation as the system reaches equilibrium after deposition [31]. In this way, PG functions as a molecular regulator that fine-tunes the balance between processability and structural stabilization. Here, the kefiran-based formulation is evaluated through quantitative filament collapse analysis, oscillatory rheology (including strain, frequency, and time sweep measurements), and microstructural examination of freeze-dried constructs. Swelling behavior and apparent mass variation under aqueous conditions are further assessed to characterize structural coherence over time. By focusing on the relationship between time-dependent viscoelastic evolution and filament stability, this study provides a detailed assessment of kefiran as an extrusion-processable, physically structured polymer system.

## 2. Materials and Methods

### 2.1. Materials

Commercial kefir grains originally cultured in whole milk were purchased from Kefiring^®^ (Montichiari, Italy) and used as the raw source for kefiran extraction. Propylene glycol (PG, ≥99.5%) was supplied by Sigma-Aldrich (Merck, Darmstadt, Germany). Ultrapure water (Milli-Q system, Millipore, Merck) was used for all preparation and purification steps.

### 2.2. Preparation of Kefiran-Based Polymer Formulation

Kefiran was extracted and purified following the procedure previously reported by Piermaria et al. [34,35], with minor adaptations in extraction temperature and purification cycles. Briefly, a weighed amount of kefir grains corresponding to 10% *w*/*v* relative to the solvent volume was dispersed in ultrapure water and heated at 90 °C for 120 min under continuous magnetic stirring. The resulting suspension was centrifuged (Thermo Scientific SL40R, Waltham, MA, USA) at 10,000× *g* for 20 min at 20 °C to remove insoluble residues and cellular components. The supernatant was collected and mixed with ethanol in a 1:1 *v*/*v* ratio to induce polysaccharide precipitation, and the mixture was stored at −20 °C overnight. The precipitated material was recovered by centrifugation (10,000× *g*, 20 min, 4 °C). The solubilization–precipitation cycle was repeated twice to improve purification. The final product was lyophilized and stored in dry form until use.

The overall extraction yield was approximately 3% relative to the initial wet mass of kefir grains. This value is consistent with previously reported yields for kefiran extracted from whole kefir grains using aqueous extraction and ethanol precipitation, as the polysaccharide represents a minor fraction of the hydrated biomass [35,36].

For formulation preparation, freeze-dried kefiran was dissolved in ultrapure water at a concentration of 6% *w*/*w* under magnetic stirring for 2 h at 70 °C in a closed vessel to minimize solvent evaporation. Propylene glycol was subsequently added as a low-molecular-weight processing aid at a kefiran-to-PG mass ratio of 4:1, based on previous optimization work on kefiran hydro-alcoholic gels [25]. After additional stirring at room temperature, a homogeneous polymer formulation with a final total polymer concentration of 4.8% *w*/*w* was obtained due to dilution upon PG addition. Before extrusion and characterization, the formulation was allowed to rest for 1 h at room temperature to promote the onset of spontaneous physical network formation, as later supported by rheological measurements.

### 2.3. Extrusion-Based Printing of Kefiran Ink

Extrusion-based printing of the polymer formulations was performed using a Rokit Invivo 4D2 system (Rokit Healthcare Inc., Seoul, Republic of Korea), firmware version 1.80. Three-dimensional models were designed using computer-aided design (CAD) software and converted into a machine-compatible format using NewCreatorK v1.57.70. The formulations were loaded into 10 mL syringes equipped with Luer-Lock connectors (internal diameter 15.5 mm) and extruded through QuantX™ blunt-tip needles (Oxnard, CA, USA) with a nominal gauge of 20 G (internal diameter 0.6 mm). Printing was carried out at room temperature (25 °C) using an extrusion speed of 4 mm s^−1^, a layer height of 0.4 mm, a flow rate set to 110% relative to the nominal extrusion value defined by the slicing software, and a fill density of 30%. Under these conditions, cylindrical constructs with a nominal diameter of 13 mm and a height of 3 mm were reproducibly fabricated. These parameters were selected to ensure continuous filament deposition and to evaluate the ability of the formulation to retain shape fidelity after extrusion.

### 2.4. Filament Collapse Test

The filament collapse test was adapted from the method originally proposed by Therriault et al. [37], in which the mid-span deflection of suspended filaments was used to evaluate the post-deposition stability of soft, extruded materials. A testing platform consisting of parallel pillars (*l* × *w* × *h* = 2.0 × 2.0 × 4.0 mm) separated by defined gap distances (1.0, 2.0, 4.0, 8.0, and 16.0 mm) was designed using computer-aided design software and fabricated using a Formlabs Form 2 stereolithography printer (Formlabs Inc., Somerville, MA, USA) with Clear Resin V4, following the manufacturer’s recommended printing and post-curing protocols. Single filaments were deposited across the pillar gaps using the same extrusion-based printing system and conditions described in the previous section. The nozzle tip was positioned at a distance of 0.3 mm above the top surface of the pillars, and the print path was extended by 10 mm beyond the last pillar to ensure steady filament formation before suspension. Filament collapse tests were performed at two defined time points: 5 min and 15 min after the addition of propylene glycol, to account for the time-dependent physical gelation and viscosity evolution of the formulation. Filament deflection was quantified by measuring the maximum vertical deflection (δ) at mid-span from still images acquired 10 s after filament deposition using FIJI/ImageJ software (version 1.54, NIH, Bethesda, MD, USA). Deflection values were normalized by half the spanning distance (*L*/2) to obtain a dimensionless deflection ratio (*δ*/*L*/2). For each condition, measurements were performed on three independent replicates and are reported as mean ± standard deviation. Normalized deflection values (*δ*/*L*/2) were plotted as a function of the half-span distance (*L*/2) to evaluate the dependence of filament stability on unsupported span length and resting time after PG addition.

### 2.5. Morphological Analysis (SEM)

The surface and internal morphology of the printed kefiran-based polymer constructs were analyzed by scanning electron microscopy (SEM) using a Phenom ProX microscope (Phenom-World, Eindhoven, The Netherlands). Observations were carried out at an accelerating voltage of 15 kV. Images were acquired without a conductive coating using the microscope’s low-vacuum mode. Before imaging, samples were freeze-dried, mounted on aluminum stubs, and fixed using conductive carbon tape. Internal cross-sections were obtained by fracturing the freeze-dried samples. SEM analysis was performed to evaluate the microstructural features of the constructs after extrusion and drying, with particular attention to filament morphology, pore architecture, and surface texture. Quantitative analysis of pore size distribution was conducted on five SEM images acquired at different magnifications for each sample, with 50 pores analyzed per sample using FIJI/ImageJ software (version 1.54, NIH, USA). Binarized SEM images were also analyzed in FIJI/ImageJ following scale calibration, grayscale conversion, and global thresholding. Apparent porosity was calculated using the area fraction of pore regions relative to the total analyzed cross-sectional area.

### 2.6. Swelling Behavior and Mass Loss

The swelling behavior of the printed kefiran-based polymer constructs was evaluated under controlled temperature conditions to assess hydration capacity and structural response in aqueous environments. Previously printed and freeze-dried samples were weighed in their dry state (*W*_0_) using a high-precision analytical balance (Sartorius Cubis II, Sartorius AG, Göttingen, Germany). The samples were then individually immersed in 24-well plates containing 1 mL of ultrapure water and incubated at 37 °C. At predefined time points (1, 2, 5, and 24 h), samples were removed from the medium, gently blotted with filter paper to remove excess surface liquid, and immediately weighed (*W_t_*). Each sample was subsequently re-immersed in the same volume of water until the subsequent measurement. The degree of swelling was calculated according to the following equation:

$$\mathrm{Swelling}\;(\%)=\frac{\left(W_{t}-W_{0}\right)}{W_{0}}\times 100$$

where *W*_0_ represents the initial dry weight, and *W_t_* represents the weight at time *t*. All experiments were performed in independent triplicates (n = 3). A 95% confidence interval for the mean swelling values was applied to evaluate the stability of the swelling plateau between 5 h and 24 h.

Long-term apparent mass loss was assessed to evaluate the structural stability of the polymer constructs under prolonged aqueous exposure. Samples previously hydrated for 24 h, corresponding to the swelling plateau and considered the condition of maximum water uptake (*W_i_*), were maintained immersed in ultrapure water at 37 °C for up to 56 days. The medium was replaced every 48 h to avoid ion accumulation and to maintain constant solution conditions. At selected time points (7, 14, 21, 28, 44, and 56 days), samples were removed, gently blotted to eliminate surface liquid, and weighed (*W*_f_).

Apparent mass loss was calculated as a percentage variation relative to the initial hydrated weight according to the following equation:

$$\mathrm{Mass}\;\mathrm{loss}\;(\%)=\frac{\left(W_{i}-W_{f}\right)}{W_{i}}\times 100$$

where *W_i_* is the weight of the hydrated sample at 24 h, and *W_f_* is the weight measured at time *t*. Results are reported as mean ± standard deviation. The data were further analyzed to identify temporal trends and to determine whether a mass-loss plateau indicative of structural stabilization was reached.

### 2.7. FTIR/ATR Analysis

Fourier transform infrared spectroscopy in attenuated total reflectance mode (FT-IR/ATR) was performed using an FT-IR/NIR Spectrum 400 spectrophotometer (PerkinElmer Inc., Wellesley, MA, USA). FT-IR/ATR spectra were acquired for pure PG, for purified kefiran immediately after extraction and lyophilization, and for printed kefiran–PG formulation after extrusion and subsequent lyophilization. Spectra were collected over the wavenumber range from 4000 to 500 cm^−1^ at standard spectral resolution. All samples were analyzed in the dry state. During acquisition, uniform pressure was applied to the sample surface to ensure consistent contact with the ATR crystal and reproducible spectral quality.

### 2.8. Rheological Characterization

The rheological behavior of the kefiran-based polymer formulation was characterized using an ARES-G2 rotational rheometer (TA Instruments, New Castle, DE, USA) equipped with a 25 mm parallel plate geometry. All measurements were performed at 25 °C, corresponding to the temperature used during extrusion printing. Before data acquisition, strain sweep tests were conducted to identify the strain range over which oscillatory measurements could be performed without inducing abrupt structural disruption of the formulation. Based on these preliminary tests, all subsequent oscillatory measurements were performed within this deformation range. To investigate the time-dependent viscoelastic evolution of the formulation after preparation, oscillatory time sweep experiments were performed by continuously monitoring the storage (*G*′) and loss (*G*″) moduli as a function of time. Measurements were carried out at a fixed angular frequency of 1 rad s^−1^ and constant temperature (25 °C), corresponding to the conditions used during extrusion printing. The selected frequency represents low-deformation conditions relevant to post-extrusion structural stabilization. The total duration of each time sweep was 30 min. The temporal evolution of *G*′ and *G*″ was used to evaluate progressive changes in viscoelastic properties associated with physical network consolidation following PG addition. Particular attention was given to the relative magnitude of the two moduli and to their divergence over time, rather than to the identification of a discrete gel point. All measurements were performed in triplicate (n = 3) to assess reproducibility.

### 2.9. Statistical Analysis

Data were analyzed using GraphPad Prism v8 (GraphPad Software, San Diego, CA, USA) and are reported as mean ± standard deviation (n = 3), unless otherwise stated. Statistical analysis was limited to swelling measurements, where a 95% confidence interval of the mean was used to assess the presence of a swelling plateau.

## 3. Results

### 3.1. Filament Collapse and Time-Dependent Printability

**Figure 1:**
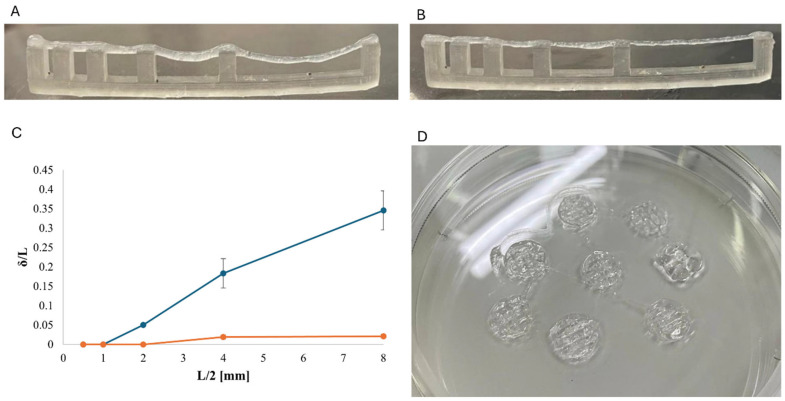
Extrusion stability and filament collapse behavior of kefiran–PG formulations: (**A**) Representative image of a suspended filament printed 5 min after PG addition, showing pronounced mid-span deflection. (**B**) Representative image of a suspended filament printed 15 min after PG addition, exhibiting reduced sagging and improved shape retention. (**C**) Quantitative analysis of filament collapse expressed as normalized mid-span deflection, *δ*/(*L*/2), plotted as a function of the half-span distance (*L*/2). Data are reported as mean ± standard deviation (n = 3); blue line = printed 5 min after PG addition and orange line = printed 15 min after PG addition. (**D**) Representative image of a printed kefiran–PG construct immediately after extrusion, demonstrating continuous filament deposition and macroscopic shape preservation.

Extrusion stability and immediate post-deposition shape retention of the kefiran–PG formulation were first evaluated by printing simple three-dimensional constructs. Under the selected printing conditions, continuous filament deposition was achieved without filament breakage or macroscopic collapse, and printed structures maintained their overall geometry immediately after extrusion (Figure 1D), indicating sufficient flow continuity and self-supporting behavior at the time of deposition.

The stability of extruded filaments was further assessed through a filament collapse test using suspended filaments spanning increasing gap distances. Representative images of filaments printed 5 min and 15 min after PG addition are shown in Figure 1A and Figure 1B, respectively. Filaments deposited 5 min after PG addition exhibited pronounced mid-span deflection, which increased with the gap size. In contrast, filaments printed after 15 min displayed markedly reduced sagging and improved shape retention, even at the largest tested spans.

Filament deformation was quantified by measuring the maximum mid-span deflection (δ) and normalizing it by half of the total spanning distance (*L*/2), yielding a dimensionless deflection ratio *δ*/(*L*/2). The normalized deflection is reported in Figure 1C as a function of the half-span length (*L*/2). For filaments printed 5 min after PG addition, *δ*/(*L*/2) increased monotonically with increasing span, reaching values of approximately 0.35 at the largest half-span. Conversely, filaments deposited after 15 min exhibited consistently low normalized deflection values (<0.03) across all tested gaps, indicating minimal gravitational collapse.

The clear separation between the two datasets highlights a strong time dependence of filament stability following PG addition.

### 3.2. Morphological Analysis (SEM)

The microstructure of the printed kefiran–PG constructs was investigated by scanning electron microscopy on fractured cross-sections and external surfaces after freeze-drying. SEM observations revealed a porous and interconnected architecture extending throughout the bulk of the constructs in the dry state.

**Figure 2:**
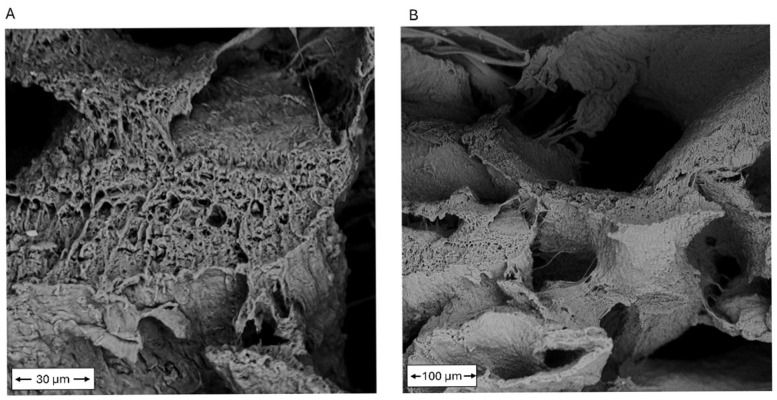
Cross-sectional SEM images of freeze-dried kefiran–PG constructs: (**A**) High-magnification image (2000×) showing the internal microtexture of pore walls, characterized by lamellar features and fine fibrillar bridges connecting adjacent domains. (**B**) Low-magnification image (500×) revealing a porous and interconnected architecture with irregular pore geometry and pore sizes ranging from several tens to hundreds of micrometers. All images were acquired on fractured cross-sections after freeze-drying.

Cross-sectional SEM images at high magnification (2000×) (Figure 2A) showed that the internal pore walls exhibited a complex microtexture characterized by thin lamellar features and fine fibrillar bridges connecting adjacent domains. At lower magnification (500×) (Figure 2B), the cross-sections revealed the presence of large, irregularly shaped pores with characteristic dimensions ranging from several tens to hundreds of micrometers, separated by continuous polymer walls forming an interconnected network. These morphological features indicate the formation of a structurally continuous polysaccharide matrix capable of maintaining integrity after solvent removal by freeze-drying. It should be noted that the observed lamellar and fibrillar features may be influenced by the freeze-drying process, which is known to induce ice-templating effects and microstructural rearrangements in hydrated polymeric systems.

**Figure 3:**
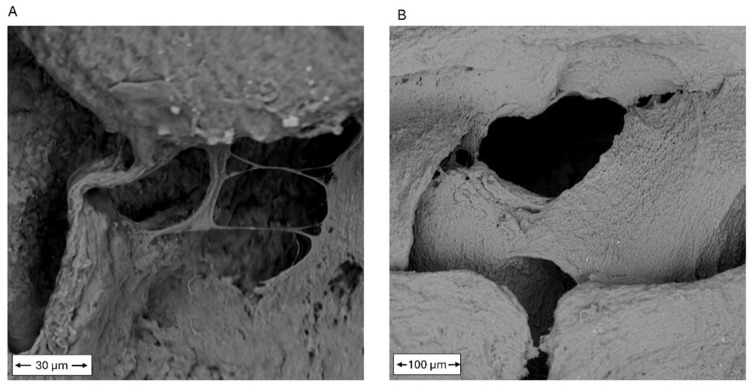
Surface SEM images of freeze-dried kefiran–PG constructs: (**A**) High-magnification image (2000×) highlighting rough pore walls and fine filamentous connections at the construct surface. (**B**) Low-magnification image (500×) showing heterogeneous and open surface pores connected to the internal pore network. The observed surface morphology is consistent with the interconnected architecture identified in the cross-sectional analysis.

Surface SEM images displayed a morphology consistent with the internal architecture observed in cross-section. At 2000× magnification (Figure 3A), the surface exhibited rough pore walls and fine filamentous connections, suggesting that the internal microstructure extends to the construct surface rather than being confined to the bulk interior. At lower magnification (500×) (Figure 3B), the surface appeared heterogeneous and porous, with open cavities directly connected to the underlying pore network, supporting the presence of a continuous porous structure in the dry state.

**Figure 4:**
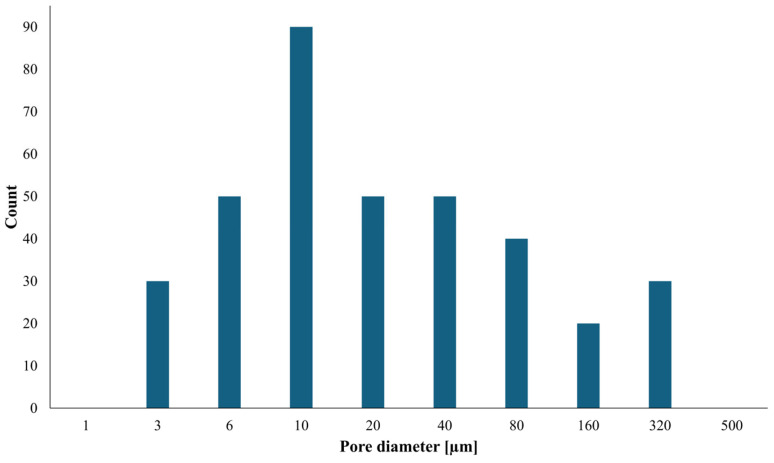
Frequency distribution of pore diameters derived from quantitative analysis of SEM images of freeze-dried kefiran–PG constructs. The distribution spans from a few micrometers to several hundred micrometers, with a predominant population in the 6–20 µm range and a modal pore diameter of approximately 10 µm. Larger pores between 40 and 160 µm, as well as sporadic cavities exceeding 300 µm, are also detected. Pore size was calculated from two-dimensional SEM micrographs using equivalent circular diameters, and values are reported per class.

Quantitative image analysis was performed on SEM micrographs to assess the pore size distribution of the freeze-dried constructs. The resulting frequency distribution (Figure 4) spans from a few micrometers to several hundred micrometers. The majority of pores fall within the micrometer range, with a predominant population between 6 and 20 µm and a modal pore diameter of approximately 10 µm. A substantial fraction of larger pores in the 40–160 µm range is also detected, together with sporadic cavities exceeding 300 µm. This pore size distribution may reflect the combined influence of extrusion-induced structuring and morphological changes associated with freeze-drying.

To estimate the porosity of the constructs in the dry state, binarized cross-sectional SEM images were analyzed to calculate the apparent two-dimensional porosity, expressed as area fraction. The resulting apparent 2D porosity was 60.9 ± 1.2%, indicating a highly porous structure. Visual inspection revealed no evident preferential pore orientation along the extrusion direction, suggesting that the microstructure is predominantly governed by ice-templating during freeze-drying rather than shear-induced alignment during processing. It should be noted that this value represents an apparent porosity derived from two-dimensional images and does not correspond to the true three-dimensional volumetric porosity of the constructs. Moreover, the measured porosity may be affected by dehydration-induced shrinkage or partial pore collapse occurring during freeze-drying. Nevertheless, the consistently observed interconnected pore architecture across different magnification levels is consistent with the observed swelling behavior upon rehydration.

### 3.3. Swelling Behavior and Apparent Mass Variation

The hydration behavior of the printed kefiran–PG constructs was investigated by monitoring swelling kinetics and apparent mass variation under aqueous conditions at 37 °C. Swelling experiments were conducted by immersing the constructs in ultrapure water and measuring their mass increase over time.

**Figure 5:**
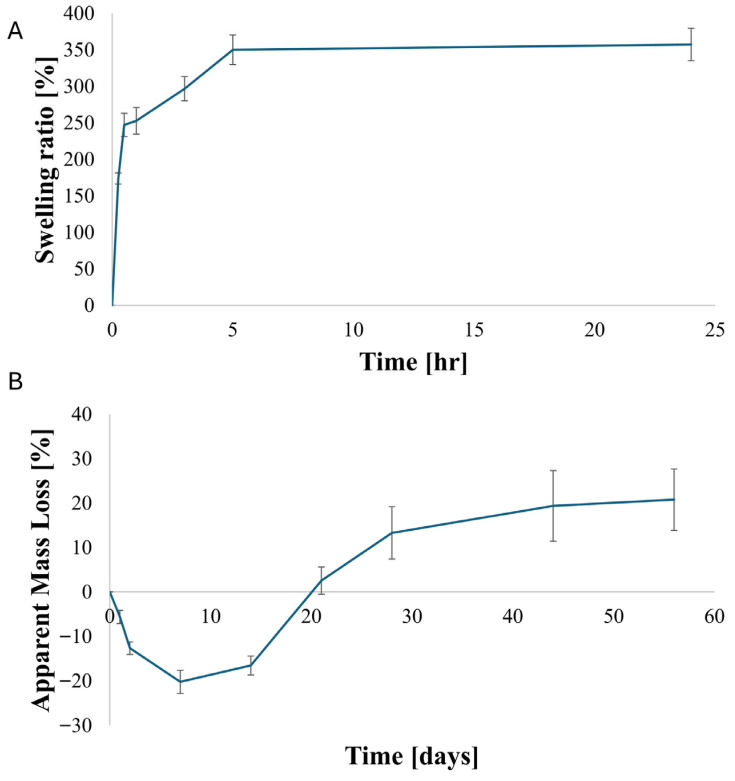
Swelling behavior and apparent mass loss of printed kefiran–PG constructs: (**A**) Swelling ratio as a function of immersion time in ultrapure water at 37 °C, showing rapid water uptake and behavior consistent with the attainment of an apparent swelling equilibrium within 5 h. (**B**) Apparent mass loss (%) of hydrated constructs during long-term incubation in water at 37 °C, showing an initial equilibration phase followed by a gradual and progressive increase in apparent mass loss up to 56 days. Data are reported as mean ± standard deviation (n = 3).

As shown in Figure 5A, the constructs exhibited a rapid swelling response during the initial hours of immersion. The swelling ratio increased to approximately 250% within the first hour and further rose to around 350% after 5 h. Beyond 5 h, no significant further increase in swelling was observed up to 24 h. The mean swelling values measured at 5 h and 24 h showed overlapping variability ranges, indicating that further swelling could not be resolved within the experimental uncertainty. Within the explored time window, the swelling behavior is therefore consistent with the attainment of an apparent swelling equilibrium.

The long-term behavior of the constructs under aqueous conditions was assessed by monitoring apparent mass variation over a period of 56 days (Figure 5B). At early time points, a slight apparent mass gain was observed, which can be attributed to ongoing equilibration and residual water uptake following initial hydration. After this equilibration phase, the constructs exhibited a gradual and time-dependent increase in apparent mass loss. Apparent mass loss reached approximately 10–15% within the first 28 days and increased to around 20% after 56 days of incubation in water at 37 °C.

The gradual increase in apparent mass loss over time suggests a progressive material erosion process without abrupt structural failure. Throughout the entire incubation period, the constructs remained macroscopically intact and could be manually handled without visible fragmentation, indicating preservation of overall structural coherence despite ongoing mass loss. This observation is qualitative in nature and reflects macroscopic integrity rather than a quantitative assessment of mechanical stability.

### 3.4. FTIR/ATR Analysis

**Figure 6:**
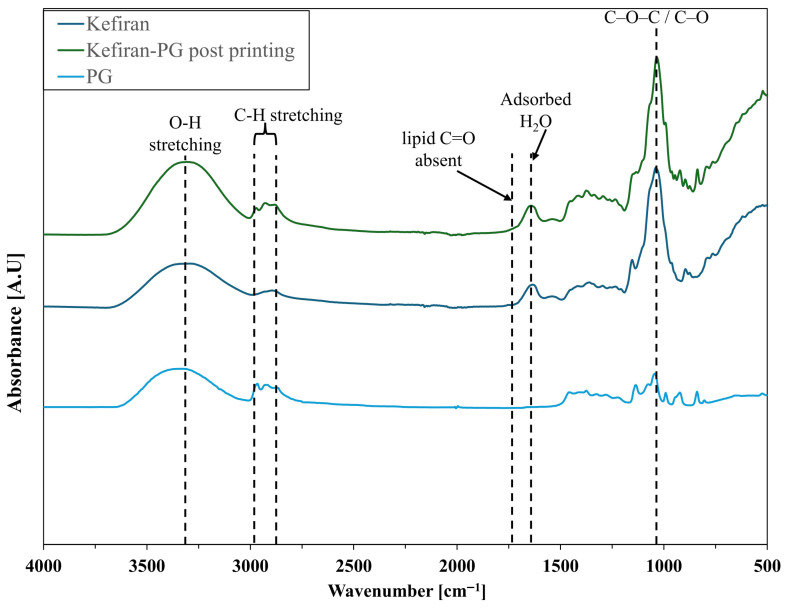
ATR–FTIR spectra of freeze-dried native kefiran (dark blue), freeze-dried printed constructs formulated from kefiran and PG (green), and pure PG (light blue), included as a spectral reference. Dashed vertical lines indicate the main absorption features discussed in the text: O–H stretching (~3350 cm^−1^), aliphatic C–H stretching (2920–2850 cm^−1^), adsorbed water bending (~1640 cm^−1^), and the C–O–C/C–O fingerprint region (~1000 cm^−1^). The absence of any absorption in the ~1735–1750 cm^−1^ region (indicated as “lipid C=O absent”) in all three spectra confirms the absence of ester-type lipid contamination. The pure PG spectrum further demonstrates that the modest C–H enhancement observed in the kefiran/PG construct arises from PG incorporation and not from lipid-derived interference. The printed construct retains the primary polysaccharide band pattern, with increased intensity in the O–H and fingerprint regions, consistent with modified hydrogen-bonding interactions and physical incorporation of PG, without evidence of new covalent functional groups.

ATR–FTIR spectra were collected on freeze-dried native kefiran, on freeze-dried printed constructs formulated from kefiran and PG, and on pure PG as a spectral reference (Figure 6). Lyophilization was performed in both cases to reduce residual water and enable comparison under comparable solid-state conditions.

The spectrum of kefiran after extraction from grains displays the characteristic features of a polysaccharide matrix. A broad absorption centered at approximately 3350 cm^−1^ is attributed to O–H stretching vibrations arising from extensive intra- and intermolecular hydrogen bonding. The bands at 2920–2850 cm^−1^ correspond to aliphatic C–H stretching modes. A weak absorption at approximately 1640 cm^−1^ is attributed to the bending vibration of residual adsorbed water retained by the hygroscopic polysaccharide matrix, as commonly reported for lyophilized carbohydrate materials [38,39,40]. In the fingerprint region, intense absorptions between 1150 and 1000 cm^−1^, with a dominant peak around 1030–1050 cm^−1^, are assigned to C–O–C glycosidic linkages and C–O stretching of the sugar rings [41].

The spectrum of the printed kefiran/PG construct retains the same overall band positions, indicating preservation of the primary polysaccharide structure after processing. However, measurable differences in relative intensities and band profiles are observed. The O–H stretching band appears broader and more intense compared to native kefiran, indicating a change in the distribution of hydrogen-bonded O–H environments. In parallel, the absorptions in the 2920–2850 cm^−1^ region are modestly enhanced. As confirmed by the reference spectrum of pure PG included in Figure 6, this enhancement is consistent with the aliphatic C–H groups of the incorporated PG molecule and is not attributable to lipid contamination: the pure PG spectrum lacks the characteristic sharp methylene doublet at ~2918/2850 cm^−1^ typical of long-chain fatty acids or triglycerides, and no ester carbonyl band near 1740 cm^−1^ is detected in any of the recorded spectra. Critically, the spectral region at ~1735–1750 cm^−1^ (diagnostic for ester carbonyl stretching in lipids) is silent in all three recorded spectra, as indicated in Figure 6, providing direct spectroscopic evidence against the presence of residual lipid contaminants [42]. The fingerprint region (1200–900 cm^−1^) exhibits increased intensity and slightly improved band definition, particularly around 1050 cm^−1^, indicating modifications in the local environment of C–O and C–O–C groups [43]. Notably, no new absorption bands were detected in regions characteristic of carbonyl formation or other covalent modifications.

### 3.5. Rheological Characterization

**Figure 7:**
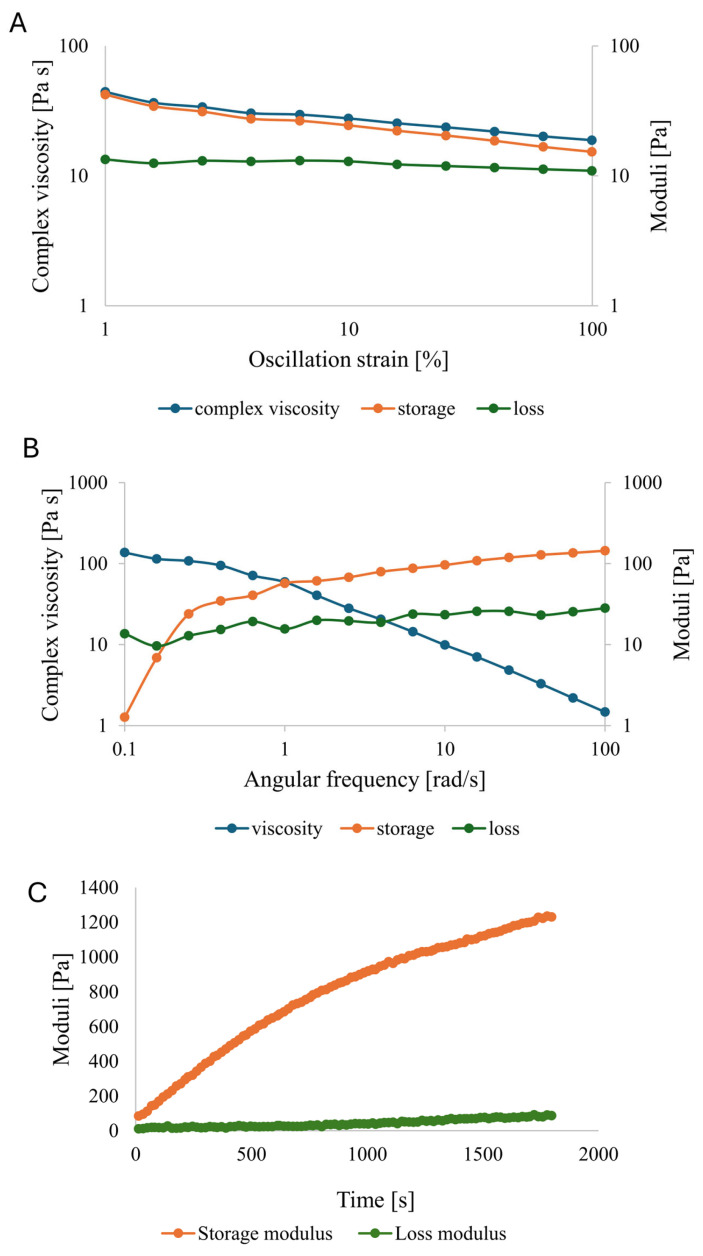
Rheological response of the kefiran–PG formulation: (**A**) Strain sweep showing the evolution of complex viscosity, storage modulus (*G*′), and loss modulus (*G*″) as a function of oscillatory strain. (**B**) Frequency sweep illustrating a frequency-dependent decrease in complex viscosity, indicative of shear-thinning-like behavior under oscillatory conditions. (**C**) Time sweep showing the temporal evolution of *G*′ and *G*″, evidencing progressive network consolidation.

The viscoelastic properties of the kefiran–PG formulation were investigated to characterize its deformation-dependent response and time-dependent structural evolution relevant to extrusion-based processing (Figure 7).

Strain sweep measurements (Figure 7A) were performed to identify the linear viscoelastic region (LVR) and to evaluate the structural response under increasing deformation. Within the low-strain regime (up to approximately 1% strain), both *G*′ and *G*″ remained nearly constant, indicating a linear viscoelastic response. Beyond this range, a gradual decrease in both moduli was observed with increasing oscillatory strain, without a sharp crossover or abrupt modulus drop. The absence of a distinct yielding point within the explored deformation window suggests a progressively weakening structure rather than a brittle network disruption.

Frequency sweep measurements (Figure 7B) showed a marked frequency dependence of the viscoelastic response. The complex viscosity decreased progressively with increasing angular frequency, displaying a frequency-dependent reduction in complex viscosity indicative of shear-thinning behavior under oscillatory conditions. In parallel, the storage modulus increased with frequency and remained comparable to or higher than the loss modulus, particularly at higher frequencies, indicating a transition toward a more elastic-dominated response at shorter timescales. Such behavior is characteristic of physically structured polymer systems governed by reversible, non-covalent interactions.

Time sweep experiments (Figure 7C) revealed a continuous increase in *G*′ throughout the measurement window, while *G*″ remained substantially lower and increased only moderately. The progressive divergence between *G*′ and *G*″ reflects a gradual increase in elastic dominance over time rather than an instantaneous gelation event. This time-dependent stiffening is consistent with a progressive viscoelastic consolidation of the formulation following PG addition and correlates with the observed improvement in filament stability.

## 4. Discussion

Microbial polysaccharides represent a distinct class of biomass-derived polymers whose physicochemical properties are defined by fermentation-driven biosynthesis [13]. Within this group, kefiran has been studied in the context of food gels and cast hydrogels, whereas its behavior under extrusion-based processing has received comparatively limited attention [44]. The present results extend existing knowledge by demonstrating that kefiran-based formulations can be tuned to satisfy the dual requirements of flow during extrusion and progressive structural stiffening after deposition, a balance that is essential for shaping soft, physically structured polymer systems.

The filament collapse analysis provides direct insight into the post-deposition response of the extruded material. The marked reduction in mid-span deflection observed between early and later deposition times indicates a time-dependent increase in resistance to gravitational deformation [45]. Comparable improvements in filament stability have been reported for physically structured polysaccharide-based inks, where structural recovery after shear occurs through reversible interactions rather than permanent cross-linking [46,47]. In the present system, the delayed stabilization observed after PG addition reflects a gradual increase in viscoelastic stiffness, as confirmed by time sweep measurements. From a processing perspective, this progressive consolidation provides a practical operational window in which extrusion can occur under moderate stresses while filament deformation remains limited after deposition.

The dry-state microstructure revealed by SEM shows a porous and interconnected architecture spanning multiple length scales. Extrusion introduces filament-level spatial heterogeneity, while solvent crystallization during lyophilization can amplify structural anisotropy through ice templating [48,49]. Therefore, the dry-state morphology should not be viewed as a direct representation of the hydrated network. Instead, the broad pore size distribution likely reflects the combined imprint of deposition geometry and phase separation during dehydration. The presence of continuous polymer walls across large cavities suggests that structural cohesion is maintained even in regions of low apparent density [50,51]. Importantly, the observed 2D porosity is compatible with rapid water ingress, indicating that macro-scale connectivity is preserved despite dehydration-induced restructuring.

Hydration behavior further clarifies network stability. Rapid swelling followed by apparent stabilization suggests that water uptake is limited not by diffusion but by structural constraints imposed by the reorganized matrix [52,53]. For kefiran systems, concentration-dependent evolution of mechanical properties under aqueous conditions has been previously reported [25], but the present results extend this framework by showing that extrusion history and post-mixing maturation influence the hydrated response indirectly through structural preconditioning. The gradual mass loss observed during prolonged incubation aligns with progressive surface erosion or partial solubilization described for physically stabilized polysaccharides [54,55]. Crucially, the absence of fragmentation indicates that cohesive interactions persist despite partial material loss. This distinction suggests that network connectivity is not solely dependent on labile surface-associated components but on a distributed non-covalent interaction framework capable of maintaining integrity over time [56].

Rheological characterization provides a coherent framework for interpreting the observed extrusion and stability behavior. The strain sweep measurements indicate a defined linear viscoelastic region followed by a gradual modulus decrease under increasing deformation, without evidence of abrupt structural collapse. Such a response is characteristic of soft, physically structured polymer systems that deform through progressive rearrangement rather than brittle failure [57,58,59]. The frequency-dependent reduction in complex viscosity reflects the viscoelastic adaptability of the formulation under increasing deformation rates, a feature commonly associated with extrusion-based manufacturing of polysaccharide systems [60,61]. Time sweep experiments further clarify the consolidation behavior of the kefiran–PG formulation. The continuous increase in storage modulus, accompanied by a comparatively smaller evolution of the loss modulus, indicates the progressive dominance of elastic contributions over time. This trend is consistent with time-dependent viscoelastic consolidation previously reported for kefiran and related microbial polysaccharides [62,63,64]. Importantly, interpreting the storage–loss modulus relationship as a descriptor of evolving viscoelastic balance, rather than as evidence of a discrete sol–gel transition, avoids overinterpretation and aligns with current rheological practice for physically assembled materials.

In this framework, ATR–FTIR provides molecular-scale information that complements the rheological and extrusion data. The preservation of band positions associated with glycosidic linkages confirms that the polysaccharide backbone remains chemically intact after processing. The absence of additional carbonyl or ester signals further supports the lack of detectable covalent modification under the applied conditions. The weak absorption at ~1640 cm^−1^ present in both the kefiran and kefiran/PG spectra is consistent with the bending mode of residual adsorbed water associated with the hygroscopic polysaccharide matrix, and its presence in kefiran alone (prior to any PG addition) confirms it is unrelated to PG incorporation or lipid contamination. The observed broadening and increased intensity of the O–H stretching band are consistent with a redistribution of hydrogen-bonded environments [65]. Similar spectral behavior has been reported in polysaccharide systems plasticized with low-molecular-weight polyols, where competitive hydrogen bonding modifies intermolecular association without altering chemical identity [66,67]. In such systems, polyols act as hydrogen-bond mediators, weakening direct polymer–polymer contacts while enabling dynamic intermolecular connectivity [68,69]. Likewise, the increased definition within the 1200–900 cm^−1^ region suggests modifications in short-range chain organization rather than changes in covalent structure. Variations in this fingerprint region have been associated with altered chain packing and local ordering in physically plasticized carbohydrate matrices [70]. When interpreted together with the progressive increase in storage modulus over time, the spectroscopic findings support a mechanism of physical reorganization in which PG participates in the hydrogen-bonding network and contributes to time-dependent viscoelastic consolidation without forming permanent cross-links.

Taken together, these results indicate that extrusion performance in kefiran–PG systems is governed by structural kinetics rather than static formulation parameters. The material evolves from a deformation-prone viscoelastic fluid to a self-supporting physically assembled network through progressive supramolecular reorganization. This mechanism distinguishes the system from ionically cross-linked or chemically cured bioinks and identifies formulation aging as a controllable design variable. While further studies are required to quantify hydrated mechanical properties and to resolve network topology under fully swollen conditions, the present work establishes a mechanistically coherent link between molecular interactions, viscoelastic evolution, and macroscopic filament stability.

## 5. Conclusions

A kefiran–propylene glycol formulation was shown to function as a time-evolving extrusion ink, in which post-mixing maturation leads to a progressive improvement in filament stability. The observed shift from deformation-prone filaments at early times to self-supporting spans after resting identifies formulation aging as a practical, chemistry-free parameter for tuning print fidelity without the need for external cross-linking steps. Based on quantitative filament collapse analysis, a post-mixing waiting time of approximately 15 min is recommended to achieve optimal shape retention, as demonstrated by the significant reduction in normalized mid-span deflection (*δ*/*L*) compared to the 5 min condition across the tested span range (*L*/2 up to 8 mm).

Across morphological and hydration analyses, the material displayed preservation of macroscopic architecture. A porous structure was observed in the freeze-dried state, and rehydration experiments demonstrated rapid swelling followed by gradual apparent mass variation under prolonged aqueous exposure. The absence of abrupt fragmentation during incubation indicates maintenance of macroscopic coherence over time, although mechanical performance in the hydrated state was not directly quantified. Consistently, ATR–FTIR analysis confirmed that processing did not alter the chemical identity of the kefiran backbone, supporting the interpretation of structural evolution occurring without covalent modification.

The combination of fully aqueous processing, time-dependent structural consolidation, and preservation of macroscopic architecture positions this kefiran-based system as a promising platform for specific application domains. In biomedical contexts, the biocompatible nature of both components, coupled with the observed porous microstructure and controlled swelling behavior, suggests potential utility in soft tissue scaffolding and drug delivery applications where gradual structural evolution is advantageous. In food-related applications, the edible nature of kefiran and propylene glycol, combined with the ability to create structured constructs through extrusion processing, opens prospects for customized food products with controlled texture and functional properties.

Rheological measurements provided a consistent physical framework for interpreting these behaviors. The progressive increase in storage modulus and the frequency-dependent viscoelastic response support a model of time-dependent viscoelastic stiffening under mild processing conditions. Together, these results establish kefiran as an extrusion-processable, physically structured polymer matrix whose properties evolve in a controllable manner after formulation. The present findings provide a foundation for further investigations aimed at assessing hydrated mechanical performance and exploring formulation modifications for targeted biomedical and food-related applications.

## Data Availability

The original contributions presented in this study are included in the article. Further inquiries can be directed to the corresponding author.

## Abbreviations

The following abbreviations are used in this manuscript:

CAD	computer-aided design
LVR	linear viscoelastic region
PG	propylene glycol
PCL	polycaprolactone
PLA	polylactic acid
SEM	scanning electron microscopy

## References

[1] Abahussain A.A., Nasr F.A., Bin Jumah A., Saravanan P., Kumar N.S., Al-Zharani M., Guganathan L., Sasikumar G., Alsalamah S.A., Ahmed Qurtam A. (2025). Toxic threats from plastic waste: Human health impacts, challenges, and policy solutions. RSC Adv..

[2] Chawla S., Varghese B.S., Chithra A., Hussain C.G., Keçili R., Hussain C.M. (2022). Environmental impacts of post-consumer plastic wastes: Treatment technologies towards eco-sustainability and circular economy. Chemosphere.

[3] Mori R. (2023). Replacing all petroleum-based chemical products with natural biomass-based chemical products: A tutorial review. RSC Sustain..

[4] Lopresti F., Capuana E., Serio G., Gentile C., Botta L. (2024). Polylactic Acid/Bamboo Leaf Extract Electrospun Mats with Antioxidant Activity for Food Packaging Applications. Antioxidants.

[5] De Luca A., Capuana E., Carbone C., Raimondi L., Carfì Pavia F., Brucato V., La Carrubba V., Giavaresi G. (2024). Three-dimensional (3D) polylactic acid gradient scaffold to study the behavior of osteosarcoma cells under dynamic conditions. J. Biomed. Mater. Res. Part A.

[6] Global Lactic Acid Market Size, Trends, Advantages, and Forecast Report—2028. https://www.marketsandmarkets.com/Market-Reports/polylacticacid-387.html.

[7] Simões C.L., Viana J.C., Cunha A.M. (2009). Mechanical properties of poly(ε-caprolactone) and poly(lactic acid) blends. J. Appl. Polym. Sci..

[8] Lopresti F., Liga A., Capuana E., Gulfi D., Zanca C., Inguanta R., Brucato V., La Carrubba V., Carfì Pavia F. (2022). Effect of Polyhydroxyalkanoate (PHA) Concentration on Polymeric Scaffolds Based on Blends of Poly-L-Lactic Acid (PLLA) and PHA Prepared via Thermally Induced Phase Separation (TIPS). Polymers.

[9] De Vega B., Dutta A., Mumtaz A., Schroeder B.C., Gerrand C., Boyd A.S., Kalaskar D.M. (2024). Comparative analysis of solvent-based and solvent-free (melting) methods for fabricating 3D-printed polycaprolactone-hydroxyapatite composite bone scaffolds: Physicochemical/mechanical analyses and in vitro cytocompatibility. Front. Bioeng. Biotechnol..

[10] de Sousa Junior R.R., dos Santos C.A.S., Ito N.M., Suqueira A.N., Lackner M., dos Santos D.J. (2022). PHB Processability and Property Improvement with Linear-Chain Polyester Oligomers Used as Plasticizers. Polymers.

[11] Tripathi N., Misra M., Mohanty A.K. (2021). Durable Polylactic Acid (PLA)-Based Sustainable Engineered Blends and Biocomposites: Recent Developments, Challenges, and Opportunities. ACS Eng. Au.

[12] Salvay A.G. (2025). Polysaccharide-Based Materials: Developments and Properties. Polymers.

[13] Mouro C., Gomes A.P., Gouveia I.C. (2024). Microbial Exopolysaccharides: Structure, Diversity, Applications, and Future Frontiers in Sustainable Functional Materials. Polysaccharides.

[14] Xue H., Feng J., Zhang B., Xie K., Liao X., Tan J. (2026). Innovative Progress of Marine Polysaccharide-Based Intelligent Food Packaging Materials: From Film-Forming Mechanisms to Intelligent Responses and Industrialization Challenges. Compr. Rev. Food Sci. Food Saf..

[15] Chadorshabi S., Mohammadi M., Shamloo E., Mahmoudzadeh M. (2025). Marine Algae-Derived Bioactives: A Sustainable Resource for the Food and Agriculture Industries. Food Front..

[16] Jim E.L., Jim E.L., Surya R., Permatasari H.K., Nurkolis F. (2025). Marine-Derived Polymers–Polysaccharides as Promising Natural Therapeutics for Atherosclerotic Cardiovascular Disease. Mar. Drugs.

[17] Bennacef C., Desobry S., Jasniewski J., Leclerc S., Probst L., Desobry-Banon S. (2023). Influence of Alginate Properties and Calcium Chloride Concentration on Alginate Bead Reticulation and Size: A Phenomenological Approach. Polymers.

[18] Temirel M., Dabbagh S.R., Tasoglu S. (2022). Shape Fidelity Evaluation of Alginate-Based Hydrogels through Extrusion-Based Bioprinting. J. Funct. Biomater..

[19] Elazzazy A.M., Baeshen M.N., Alasmi K.M., Alqurashi S.I., Desouky S.E., Khattab S.M.R. (2025). Where Biology Meets Engineering: Scaling Up Microbial Nutraceuticals to Bridge Nutrition, Therapeutics, and Global Impact. Microorganisms.

[20] Khan R., Shah M.D., Shah L., Lee P.C., Khan I. (2022). Bacterial polysaccharides—A big source for prebiotics and therapeutics. Front. Nutr..

[21] Marin M.M., Gifu I.C., Pircalabioru G.G., Albu Kaya M., Constantinescu R.R., Alexa R.L., Trica B., Alexandrescu E., Nistor C.L., Petcu C. (2023). Microbial Polysaccharide-Based Formulation with Silica Nanoparticles; A New Hydrogel Nanocomposite for 3D Printing. Gels.

[22] Yermagambetova A., Tazhibayeva S., Takhistov P., Tyussyupova B., Tapia-Hernández J.A., Musabekov K. (2024). Microbial Polysaccharides as Functional Components of Packaging and Drug Delivery Applications. Polymers.

[23] Radhouani H., Gonçalves C., Maia F.R., Oliveira J.M., Reis R.L. (2018). Kefiran biopolymer: Evaluation of its physicochemical and biological properties. J. Bioact. Compat. Polym..

[24] Dentice Maidana S., Argañaraz Aybar J.N., Albarracin L., Imamura Y., Arellano-Arriagada L., Namai F., Suda Y., Nishiyama K., Villena J., Kitazawa H. (2024). Modulation of the Gut-Lung Axis by Water Kefir and Kefiran and Their Impact on Toll-like Receptor 3-Mediated Respiratory Immunity. Biomolecules.

[25] Sabatino M.A., Pavia F.C., Rigogliuso S., Giacomazza D., Ghersi G., La Carrubba V., Dispenza C. (2020). Development of injectable and durable kefiran hydro-alcoholic gels. Int. J. Biol. Macromol..

[26] Blandón L.M., Islan G.A., Castro G.R., Noseda M.D., Thomaz-Soccol V., Soccol C.R. (2016). Kefiran-alginate gel microspheres for oral delivery of ciprofloxacin. Colloids Surf. B Biointerfaces.

[27] Mukai T., Watanabe N., Toba T., Itoh T., Adachi S. (1991). Gel-Forming Characteristics and Rheological Properties of Kefiran. J. Food Sci..

[28] Schwab A., Levato R., D’Este M., Piluso S., Eglin D., Malda J. (2020). Printability and Shape Fidelity of Bioinks in 3D Bioprinting. Chem. Rev..

[29] Tarassoli S.P., Jessop Z.M., Jovic T., Hawkins K., Whitaker I.S. (2021). Candidate Bioinks for Extrusion 3D Bioprinting—A Systematic Review of the Literature. Front. Bioeng. Biotechnol..

[30] Radhouani H., Bicho D., Gonçalves C., Maia F.R., Reis R.L., Oliveira J.M. (2019). Kefiran cryogels as potential scaffolds for drug delivery and tissue engineering applications. Mater. Today Commun..

[31] Ghasemlou M., Khodaiyan F., Oromiehie A. (2011). Physical, mechanical, barrier, and thermal properties of polyol-plasticized biodegradable edible film made from kefiran. Carbohydr. Polym..

[32] Vieira M.G.A., Da Silva M.A., Dos Santos L.O., Beppu M.M. (2011). Natural-based plasticizers and biopolymer films: A review. Eur. Polym. J..

[33] Talja R.A., Helén H., Roos Y.H., Jouppila K. (2007). Effect of various polyols and polyol contents on physical and mechanical properties of potato starch-based films. Carbohydr. Polym..

[34] Piermaria J.A., de la Canal M.L., Abraham A.G. (2008). Gelling properties of kefiran, a food-grade polysaccharide obtained from kefir grain. Food Hydrocoll..

[35] Piermaria J.A., Pinotti A., Garcia M.A., Abraham A.G. (2009). Films based on kefiran, an exopolysaccharide obtained from kefir grain: Development and characterization. Food Hydrocoll..

[36] Lopresti F., Campora S., Tirri G., Capuana E., Carfì Pavia F., Brucato V., Ghersi G., La Carrubba V. (2021). Core-shell PLA/Kef hybrid scaffolds for skin tissue engineering applications prepared by direct kefiran coating on PLA electrospun fibers optimized via air-plasma treatment. Mater. Sci. Eng. C.

[37] Therriault D., White S.R., Lewis J.A. (2007). Rheological behavior of fugitive organic inks for direct-write assembly. Appl. Rheol..

[38] Pop C., Apostu S., Rotar A.M., Semeniuc C.A., Sindic M., Mabon N. (2013). FTIR spectroscopic characterization of a new biofilm obtained from kefiran. J. Agroaliment. Process. Technol..

[39] Piermaria J., Bosch A., Pinotti A., Yantorno O., Garcia M.A., Abraham A.G. (2011). Kefiran films plasticized with sugars and polyols: Water vapor barrier and mechanical properties in relation to their microstructure analyzed by ATR/FT-IR spectroscopy. Food Hydrocoll..

[40] Pop C.R., Coldea T.E., Salanţă L.C., Nistor A.L., Borşa A., Fărcaș A.C., Florian V.C., Rotar A.M. (2021). The Effect of Extraction Conditions on the Barrier and Mechanical Properties of Kefiran Films. Coatings.

[41] Eda Ondul K., Mahmut I. (2021). Physicochemical Properties of Polysaccharide Kefiran Isolated from Kefir Grains Biomass. Int. J. Biotechnol. Biomater. Eng..

[42] Ledniowska K., Nosal-Kovalenko H., Janik W., Krasuska A., Stańczyk D., Sabura E., Bartoszewicz M., Rybak A. (2022). Effective, Environmentally Friendly PVC Plasticizers Based on Succinic Acid. Polymers.

[43] Hong S.H., Cho Y., Kang S.W. (2021). Formation of Water-Channel by Propylene Glycol into Polymer for Porous Materials. Membranes.

[44] Ghanbarzadeh B., Almasi H., Entezami A.A. (2011). Development and characterisation of a new biodegradable edible film made from kefiran, an exopolysaccharide obtained from kefir grains. Food Chem..

[45] Ribeiro A., Blokzijl M.M., Levato R., Visser C.W., Castilho M., Hennink W.E., Vermonden T., Malda J. (2018). Assessing bioink shape fidelity to aid material development in 3D bioprinting. Biofabrication.

[46] Carvalho D.N., Dani S., Sotelo C.G., Pérez-Martín R.I., Reis R.L., Silva T.H., Gelinsky M. (2023). Assessing non-synthetic crosslinkers in biomaterial inks based on polymers of marine origin to increase the shape fidelity in 3D extrusion printing. Biomed. Mater..

[47] Bharadwaj T., Chrungoo S., Verma D. (2023). Development of a novel thermogelling PEC-based ECM mimicking nanocomposite bioink for bone tissue engineering. J. Biomater. Sci. Polym. Ed..

[48] Grenier J., Duval H., Barou F., Lv P., David B., Letourneur D. (2019). Mechanisms of pore formation in hydrogel scaffolds textured by freeze-drying. Acta Biomater..

[49] Lee S.C., Gillispie G., Prim P., Lee S.J. (2020). Physical and Chemical Factors Influencing the Printability of Hydrogel-based Extrusion Bioinks. Chem. Rev..

[50] Baccile N., Messaoud G., Zinn T., Fernandes F. (2019). Soft lamellar solid foams from ice-templating of self-assembled lipid hydrogels: Organization drives the mechanical properties. Mater. Horizons.

[51] Grenier J., Duval H., Lv P., Barou F., Le Guilcher C., Aid R., David B., Letourneur D. (2022). Interplay between crosslinking and ice nucleation controls the porous structure of freeze-dried hydrogel scaffolds. Biomater. Adv..

[52] Azimzadeh B., Martínez C.E. (2025). Dynamic Sorption and Interfacial Assembly of Polysaccharide on Hydrophobic vs Hydrophilic Surfaces. ACS Earth Space Chem..

[53] Marelli F., Pontoriero D., Antonini C., Tagliaro I. (2025). Water-polysaccharide interactions and their properties in freezing conditions. Carbohydr. Polym..

[54] Cardoso M.J., Caridade S.G., Costa R.R., Mano J.F. (2016). Enzymatic Degradation of Polysaccharide-Based Layer-by-Layer Structures. Biomacromolecules.

[55] Fuoco T., Cuartero M., Parrilla M., García-Guzmán J.J., Crespo G.A., Finne-Wistrand A. (2021). Capturing the Real-Time Hydrolytic Degradation of a Library of Biomedical Polymers by Combining Traditional Assessment and Electrochemical Sensors. Biomacromolecules.

[56] Ferraz J., Pollini M.F.O., Cardoso V.M.B., Nunes S., Chorilli M., Roque-Borda C.A., Meneguin A.B. (2025). Evaluating the Impact of High-Esterified Pectin on Retrogradation and Film-Forming Properties of High-Amylose Starch. Polysaccharides.

[57] Joshi Y.M. (2025). Linear viscoelasticity of physically aging soft glassy (Thixotropic) materials. Curr. Opin. Colloid Interface Sci..

[58] Dabbaghi M., Namjoshi S., Panchal B., Grice J.E., Prakash S., Roberts M.S., Mohammed Y. (2021). Viscoelastic and Deformation Characteristics of Structurally Different Commercial Topical Systems. Pharmaceutics.

[59] Fernandes R.R., Andrade D.E.V., Franco A.T., Negrão C.O.R. (2017). The yielding and the linear-to-nonlinear viscoelastic transition of an elastoviscoplastic material. J. Rheol..

[60] Mohamed Yunus R.A., Parisi D. (2024). Scaling Laws in Polysaccharide Rheology: Comparative Analysis of Water and Ionic Liquid Systems. Biomacromolecules.

[61] Montoya J., Medina J., Molina A., Gutiérrez J., Rodríguez B., Marín R. (2021). Impact of viscoelastic and structural properties from starch-mango and starch-arabinoxylans hydrocolloids in 3D food printing. Addit. Manuf..

[62] Exarhopoulos S., Raphaelides S.N., Kontominas M.G. (2018). Flow behavior studies of kefiran systems. Food Hydrocoll..

[63] Piermaría J., Bengoechea C., Abraham A.G., Guerrero A. (2016). Shear and extensional properties of kefiran. Carbohydr. Polym..

[64] Stojkov G., Niyazov Z., Picchioni F., Bose R.K. (2021). Relationship between structure and rheology of hydrogels for various applications. Gels.

[65] Knop S., Jansen T.L.C., Lindner J., Vöhringer P. (2011). On the nature of OH-stretching vibrations in hydrogen-bonded chains: Pump frequency dependent vibrational lifetime. Phys. Chem. Chem. Phys..

[66] Sharma M., Mondal D., Mukesh C., Prasad K. (2013). Self-healing guar gum and guar gum-multiwalled carbon nanotubes nanocomposite gels prepared in an ionic liquid. Carbohydr. Polym..

[67] Srinivasa P.C., Ramesh M.N., Tharanathan R.N. (2007). Effect of plasticizers and fatty acids on mechanical and permeability characteristics of chitosan films. Food Hydrocoll..

[68] Stetsyshyn Y., Ohar H., Budkowski A., Lazzara G. (2025). Molecular Design and Role of the Dynamic Hydrogen Bonds and Hydrophobic Interactions in Temperature-Switchable Polymers: From Understanding to Applications. Polymers.

[69] Li J., Chen J., An L., Yuan X., Yao L. (2020). Polyol and sugar osmolytes can shorten protein hydrogen bonds to modulate function. Commun. Biol..

[70] Lu H., Ma R., Chang R., Tian Y. (2021). Evaluation of starch retrogradation by infrared spectroscopy. Food Hydrocoll..

